# Computational Methods for Modification of Metabolic Networks

**DOI:** 10.1016/j.csbj.2015.05.004

**Published:** 2015-05-28

**Authors:** Takeyuki Tamura, Wei Lu, Tatsuya Akutsu

**Affiliations:** Bioinformatics Center, Institute for Chemical Research, Kyoto University, Gokasho, Uji, Kyoto 6110011, Japan,

**Keywords:** Metabolic network, Constraint-based programming, Flux balance analysis, Elementary mode, Boolean model, Overfitting

## Abstract

In metabolic engineering, modification of metabolic networks is an important biotechnology and a challenging computational task. In the metabolic network modification, we should modify metabolic networks by newly adding enzymes or/and knocking-out genes to maximize the biomass production with minimum side-effect. In this mini-review, we briefly review constraint-based formalizations for Minimum Reaction Cut (MRC) problem where the minimum set of reactions is deleted so that the target compound becomes non-producible from the view point of the flux balance analysis (FBA), elementary mode (EM), and Boolean models. Minimum Reaction Insertion (MRI) problem where the minimum set of reactions is added so that the target compound newly becomes producible is also explained with a similar formalization approach. The relation between the accuracy of the models and the risk of overfitting is also discussed.

## 1 Introduction

A metabolic network represents relations between chemical reactions and compounds in a cell of organisms. Although much knowledge about metabolic networks is available in public databases and references, we often have to modify metabolic networks in various situations. For example, in metabolic engineering, we should modify metabolic networks by newly adding enzymes or/and knocking-out genes to maximize the biomass production with minimum side-effect. The former and latter correspond to adding and deleting chemical reactions, respectively, in a metabolic network. For another example, when we reconstruct a genome-scale metabolic network from a newly determined DNA sequence, the reconstructed metabolic network may need some modification to be consistent with the existing knowledge. Thus, in metabolic network modification, we often add or/and delete reactions so that specified constraints are satisfied.

Although there may exist various modification problems, we focus on the following two major problems in this mini-review: (i) *Minimum Reaction Cut* (MRC) problem: delete the minimum set of reactions so that the target compound becomes non-producible, and (ii) *Minimum Reaction Insertion* (MRI) problem: add the minimum set of reactions so that the target compound newly becomes producible. It should be noted that, for most cases, a target compound can be replaced by a set of target compounds in a straight-forward manner. In order to solve these problems, three mathematical models have been utilized: flux balance analysis (FBA) model, elementary mode (EM) model, and Boolean model. In this mini-review, we explain these three models in the context of MRC, and briefly review MRI.

**Fig. 1:**
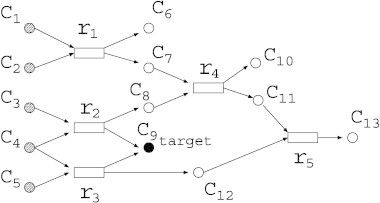
An example of a metabolic network. Rectangles and circles represent chemical reactions and compounds, respectively.

Before explaining details of each model, we briefly explain MRC. Suppose that a metabolic network of Fig. 1 is given. Rectangles and circles represent reactions and compounds, respectively. {*c*_1_,…,*c*_13_} is a set of compounds, and {*r*_1_,…,*r*_5_} is a set of reactions. For example, reaction *r*_1_ has the substrates (reactants) {*c*_1_, *c*_2_}, and products {*c*_6_, *c*_7_}. If either indegree (the number of input nodes) or outdegree (the number of output nodes) of a compound node is 0, it is called an *external node*. {*c*_1_, *c*_2_, *c*_3_, *c*_4_, *c*_5_, *c*_6_, *c*_9_, *c*_10_, *c*_13_} is a set of external nodes in Fig. 1, and the external nodes consist of *source nodes* and *sink nodes*. Compound nodes with indegree 0 are called source nodes and are assumed to be supplied by the external environment. {*c*_1_, *c*_2_, *c*_3_, *c*_4_, *c*_5_} are source nodes. On the other hand, compound nodes with outdegree 0 are called sink nodes. {*c*_6_, *c*_9_, *c*_10_, *c*_13_} are sink nodes. *Target nodes* are chosen from sink nodes. In Fig. 1. {*c*_9_} is chosen as a target node.

For example, in MRC, the solution in the Boolean model is deleting {*r*_2_, *r*_3_} because *c*_9_ is produced only from *r*_2_ or *r*_2_. However, in the EM and FBA models, if there is a chemical reaction “*A* + *B* → *C* + *D*”, C and D should also exist for the reaction to take place, in addition to A and B, because steady states are assumed (see latter sections for details of EM and FBA models). Therefore, deletion of any single reaction is the solution of MRC in the FBA and EM models since all reactions must take place at a time if some reaction takes place.

## 2 Flux Balance Analysis-based Method

Flux balance analysis (FBA) is a constraint-based mathematical framework using the stoichiometry of a given metabolic network. In many cases, FBA is used to optimize a biologically relevant objective function to identify optimal flux distributions [19,32]. In FBA, the state of the whole metabolic network is represented by fluxes for all reactions, and the sum of incoming fluxes must be equal to the sum of outgoing fluxes for each compound, where fluxes may be weighted according to the stoichiometry coefficients.

For MRC and MRI in the FBA model, in addition to the objective function in the standard FBA, the number of added or deleted reactions should also be taken into account. Furthermore, we may need to consider two objectives: cellular objective and bioengineering objective.

**Fig. 2:**
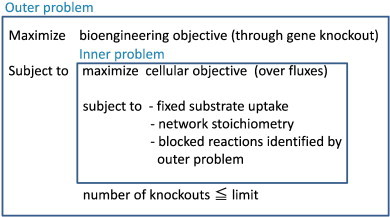
A framework of the bilevel programming [2].

In order to identify gene knockout strategies for microbial strain optimization under such a complex situation, a bilevel programming framework was introduced in [2] in which there are outer and inner optimization problems as shown in Fig. 2. The outer problem optimizes the bioengineering objective, whereas the inner problem optimizes the cellular objective.

Here, we consider MRC under the bilevel programming framework. Let *v_target_* denote the flux of the reaction that produces the target compound. Our purpose is to find the minimum number of reactions deletion of which always makes *v_target_* = 0. Then, MRC in the FBA model can be formalized as follows by starting with *K* = 0, and increment *K* by 1 until *v_target_* = 0 is obtained, where *K* is the upper limit of the number of deleted reactions.

$$\underset{s_{j}}{\mathrm{Maximize}}\;-v_{\mathrm{target}}$$

subject to

$$\underset{v_{j}}{\mathrm{Maximize}}\;v_{\mathrm{target}}$$

subject to

$$\begin{array}{l} \sum_{{}_{j}}S_{ij}\cdot v_{j}=0,\forall i\in I,\\ LB_{j}\cdot s_{j}\leq v_{j}\leq UB_{j}\cdot s_{j},\forall j\in J,\\ s_{j}\in \left\{0,1\right\},\forall j\in J,\\ \sum_{j\in J}\left(1-s_{j}\right)\leq K\text{,} \end{array}$$

where *s_j_* is a 0–1 variable, *s_ij_* is a stoichiometry matrix for the *i*th compound and *j*th reaction, *v_j_* (*j* = 1,…, *n*) is a flux vector, *I* is a set of compounds, *J* is a set of reactions, and *LB_j_* and *UB_j_* are the lower and upper bounds of *v_j_* (*j* = 1,…, *n*), respectively. *s_j_* represents whether *j*th reaction is knocked-out, where *s_j_* = 0 indicates that *j*th reaction is knocked-out since *v_j_* is forced to be 0.

In the above, we used the same function (but with different signs) as the objective functions in outer and inner optimization problems. However, there are various versions of the problem setting based on objective functions for the inner problem and the outer problem.

For example, the minimization of metabolic adjustment method (MOMA) minimizes the difference between the wild and the knocked-out flows [25]. In the flux variability analysis (FVA), both the maximum and minimum values of the objective function are calculated, and the range of them is accounted for [26]. OptKnock maximizes the bioengineering objective in the outer problem, and the cellular objective in the inner problem [2], where the upper bound of the number of removed reactions is given as in the above. On the other hand, RobustKnock maximizes the minimal possible rate of the bioengineering objective in the outer max–min problem, while the cellular objective is maximized in the inner min–max structure [30].

## 3 Elementary Mode-based Method

**Fig. 3:**
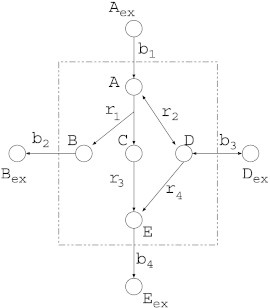
An example of a metabolic network, where reaction nodes are omitted. Elementary modes (EMs) of this network is shown in Table 1.

An elementary mode (EM) represents a feasible and balanced (steady-state) flux distribution of the network [24,23]. It must be minimal with respect to utilized reactions (enzymes). Suppose that a metabolic network of Fig. 3 is given, where reaction nodes are omitted. {*A*_*ex*_, *B*_*ex*_, *C*_*ex*_, *D*_*ex*_} is a set of external compounds. In this network, there are 5 EMs, which are shown in Table 1. Although all values in Table 1 are either 0 or 1, any real number is allowed according to the coefficients of chemical reaction formula.

The most standard version of MRC in the EM model is formalized as a minimal cut set (MCS) problem by [7]. MCS is a minimal set of reactions in the network whose inactivation leads to a failure in certain network functions. EMs and MCSs can be calculated by their developed software tools called FluxAnalyzer and CellNetAnalyzer [9,8]. Because MCS can induce side effects disabling desired functionalities, constraint MCSs (cMCSs) have been proposed, which generalize MCSs and allow for the additional definition of a set of desired modes [3]. In cMCSs, a minimum number must be preserved for the desired modes.

Once EMs are given, MRC in the EM model (MCS) can be formalized by Integer Linear Programming (ILP) [17]. Suppose that the objective is to suppress production of *E_ex_* in the metabolic network of Fig. 3 by knocking-out the minimum number of reactions. To this end, it is enough to inactivate EMs including *b*_4_, which can be represented as:

$$\mathrm{Maximize}\;r_{1}+r_{2}+r_{3}+r_{4}$$

subject to

$$\begin{array}{l} \;r_{1}\wedge r_{3}=0,\\ \;r_{4}=0,\\ r_{1}\wedge r_{2}\wedge r_{3}=0,\\ \;r_{2}\wedge r_{4}=0\text{,} \end{array}$$

where all variables are binary, and *x* ∧ *y* denotes the logical “AND” of *x* and *y* (i.e., *x* ∧ *y* = 1 if and only if *x* = 1 and *y* = 1). The Boolean constraints in the above are converted into the following linear inequalities.

$$\begin{array}{l} \;r_{1}+r_{3}\leq 1,\\ \;r_{4}=0,\\ r_{1}+r_{2}+r_{3}\leq 2,\\ \;r_{2}+r_{4}\leq 1\text{.} \end{array}$$

## 4 Boolean Model-based Method

In the Boolean model, all reaction and compound nodes are assigned either 0 or 1. If 1 is assigned, it means that the compound is producible, or the reaction can take place. On the other hand, if 0 is assigned, it means that the compound is not producible or the reaction cannot take place.

Moreover, reaction and compound nodes are represented by logical “AND” and “OR” functions, respectively. For example, in Fig. 1, *r*_1_ represents a chemical reaction “*c*_1_ + *c*_2_ → *c*_6_ + *c*_7_”. In the Boolean model, for *r*_1_ to take place, both *c*_1_ and *c*_2_ are necessary. Therefore, the condition of *r*_1_ is represented by *r*_1_ = *c*_1_ ∧ *c*_2_. Similarly, *c*_9_ is producible if either *r*_2_ or *r*_3_ takes place. Therefore, the condition of *c*_9_ is represented by *c*_9_ = *r*_2_ ∨ *r*_3_.

For MRC in the Boolean model, the Boolean reaction cut (BRC) problem has one of the most standard problem settings. In BRC, the number of deleted reactions is minimized to make the target compounds non-producible, and an ILP-based method for solving it was developed in [29].

Another standard problem setting is to minimize the side effect instead of the number of deleted reactions. The Optimal enzyme drug target identification algorithm based on metabolic networks (OPMET) was developed in [28]. OPMET identifies the optimal enzyme combination whose inhibition achieves the required effect of eliminating a given target set of compounds, while incurring minimal side-effects.

As MRC in the FBA model can be formalized by the bilevel programming with the inner and outer problems, MRC in the Boolean model also has such two layers of problems. Because each set of deleted reactions can have multiple 0/1 assignments which satisfies all Boolean constrains, some objective function should be optimized in the inner problem even in the Boolean model. This is necessary especially for properly accounting for the effect of directed cycles in metabolic networks. For this purpose, [29] introduced the notion of maximal valid assignment (MaxVA), where MaxVA is a 0/1 assignment that is maximal with respect to the number of 1s, when a set of deleted reaction is given.

In the above problem settings, the main desired effects and side non-desired effects are considered in a single metabolic network. A reasonable extension is to consider them in multiple networks. [17] developed an ILP-based method for the minimum knockout for multiple metabolic network problem (MKMN). In MKMN, when a set of source compounds and a set of target compounds are given, we must find the minimum set of reactions whose knockout ensures that the target compounds are not producible in *N*_1_, but are producible in *N*_2_.

In ILP, every constraint must be represented by linear equations or inequalities. Boolean constraints can be transformed into linear equations or inequalities as follows.

LP1 [29]: Since the Boolean “AND” relation *y* = *x*_1_ ∧ *x*_2_ ∧ ⋯ ∧ *x*_*k*_ can be converted into $\left(y\vee \bar{x_{1}}\vee \bar{x_{2}}\vee \cdots \vee \bar{x_{k}}\right)\wedge \left(\bar{y}\vee x_{1}\right)\wedge \left(\bar{y}\vee x_{2}\right)\wedge \cdots \wedge \left(\bar{y}\vee x_{k}\right)=1$, it can be represented by the following linear inequalities:

$$\begin{array}{l} y+\left(1-x_{1}\right)+\left(1-x_{2}\right)+\cdots +\left(1-x_{k}\right)\geq 1,\\ \;\left(1-y\right)+x_{1}\geq 1,\\ \;\left(1-y\right)+x_{2}\geq 1,\\ \;\cdots \\ \;\left(1-y\right)+x_{k}\geq 1\text{,} \end{array}$$

where all variables are binary.

Similarly, as the Boolean “OR” relation *y* = *x*_1_ ∨ *x*_2_ ∨ ⋯ ∨ *x*_*k*_ can be converted into $\left(\bar{y}\vee x_{1}\vee x_{2}\vee \cdots \vee x_{k}\right)\wedge \left(y\vee \bar{x_{1}}\right)\wedge \left(y\vee \bar{x_{2}}\right)\wedge \cdots \wedge \left(y\vee \bar{x_{k}}\right)=1$, it can be represented by the following linear inequalities:

$$\begin{array}{l} \left(1-y\right)+x_{1}+x_{2}+\cdots +x_{k}\geq 1,\\ \;y+\left(1-x_{1}\right)\geq 1,\\ \;y+\left(1-x_{2}\right)\geq 1,\\ \;\cdots \\ \;y+\left(1-x_{k}\right)\geq 1\text{,} \end{array}$$

where all variables are binary.

LP2 [1]: Another type of linear function representation of Boolean functions is as follows: The Boolean “AND” can be represented by the following linear inequalities:

$$\begin{array}{l} ky\leq x_{1}+x_{2}+\ldots +x_{k},\\ y\geq x_{1}+\ldots +x_{k}-\left(k-1\right)\text{,} \end{array}$$

where all variables are binary.

Similarly, the Boolean “OR” can be represented by the following linear inequalities:

$$\begin{array}{l} ky\geq x_{1}+x_{2}+\ldots +x_{k},\\ y\leq x_{1}+\ldots +x_{k}\text{,} \end{array}$$

where all variables are binary.

To calculate the MaxVA, using the notion of time is convenient in the ILP formalization [29]. However, in a naive algorithm, the number of variables is *O*((*m* + *n*)^2^), where *m* and *n* are the numbers of compounds and reactions, respectively. Because, the computational time for solving ILP is often exponential for the number of variables, the naive method cannot be applied for large scale networks.

To handle this problem, [29] developed an feedback vertex set (FVS)-based method. An FVS is a set of nodes whose removal makes a network acyclic. They formalized the MRC in the Boolean model by ILP in which the number of variables is *O*(*f*(*m* + *n* + *f*)), where *f* is the size of the FVS.

The problems formalized by ILP can also be solved by SAT-based methods since both are NP-complete problems, where SAT denotes the Boolean satisfiability problem. MRC may also be represented as an abduction problem if it is formalized by a logic programming-based method. Meta-level abduction is a method of abducting missing rules to account for observations. [5] showed that meta-level abduction can consistently produce both positive and negative causal relations. [31] developed an inductive logic programming approach to estimate possible reaction states. Their method finds hypotheses that logically explains the causal relations. Because the reaction states correspond to which reactions are active, the problem setting may correspond to MCS in the Boolean model.

## 5 Minimum Reaction Insertion

MRC problems are for finding reaction deletion strategies to satisfy given constrains. Different from MRC, the minimum reaction insertion (MRI) problems are for finding reaction addition strategies to satisfy constraints.

**Fig. 4:**
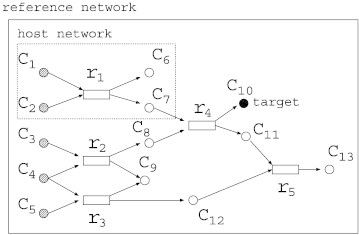
An example of a metabolic network for minimum reaction insertion (MRI) problem. The area of the dotted line is a host network and initially available. We should add the minimum set of reactions so that the target compound becomes producible.

In the given network of MRI, reactions are classified into the currently available part and the currently non-available part. We call the former and latter a host network and a reference network, respectively. In Fig. 4, only {*r*_1}_ belongs to the host network, whereas none of {*r*_2_, *r*_3,_ *r*_4,_ *r*_5_} belongs to the host network. Suppose that {*c*_1_, *c*_2_, *c*_3_, *c*_4_, *c*_5_} is a set of source nodes, {*c*_6_, *c*_9_, *c*_10_, *c*_13_} is a set of sink nodes, and *c*_10_ is a target node. In the Boolean model, adding {*r*_2_, *r*_4_} is the solution, whereas adding {*r*_2_, *r*_3,_ *r*_4,_ *r*_5_} is the solution in the FBA model.

When a metabolic network is newly reconstructed from a DNA sequence, a host network is often constructed according to the ortholog information of DNAs. However, this network often has gaps, and some of necessary compounds are not producible in this initial model. In such a case, gaps are often found and filled by FBA-based simulations. GapFind identifies non-producible metabolites based on the initial FBA model, and GapFill fills these gaps by the minimum set of additional reactions [10]. GapFill utilizes a customized multi-organism database that restores the connectivity of these metabolites to the parent network.

[16] developed a software tool, minRect, for solving MRI in the Boolean model. They call the parent network and multi-organism database as the host network and reference network, respectively. In the inner problem of MRI in the Boolean model, the notion of the minimal valid assignment is employed in order to account for the effect of directed cycles instead of the maximal valid assignment, since MRC and MRI are complementary problems.

Cell growth rate and gene essentiality are also utilized for the modification of metabolic networks. GrowMatch identifies the gaps based on the inconsistencies of cell growth rates between the simulation results on the model and the biological experiment results [11].

## 6 Discussion

### 6.1 Comparison Among the FBA, EM, and Boolean-based Models

In this article, we have briefly reviewed studies on MRC and MRI problems in the FBA, EM and Boolean-based models. The FBA and EM models can be classified into flow-based models. The flow-based models can realize more detailed simulation, because they account for the chemical reaction coefficients, the upper and lower bounds of each flux, and the steady state. Therefore, if the purpose of the research is to construct an exact model which meets the data obtained in biological experiments, the flow-based models are better than the Boolean model.

However, at the same time, this high performance of the flow models includes the risk of the overfitting. Because knowledge and data about metabolic networks are not yet perfect, an exact model may be useful only for the data used to reconstruct the model. Although the Boolean model is less detailed than the flow-based models, it is considered to be more robust from this point of view.

For example, in MRC of Fig. 1, deleting *r*_5_ is one of the optimal solution of the flow models. However, if there is an unknown reaction *r*_6_ whose substrates are *c*_8_ and *c*_12_ and products are *c*_10_ and *c*_13_, then *c*_9_ is still producible even if *r*_5_ is deleted. This shows that the flow-based models are less robust for the lack of information in the downstream of a flux. On the other hand, the optimal solution of the Boolean model is to delete {*r*_2_, *r*_3_}. Even if there is the hidden reaction *r*_6_, deleting {*r*_2_, *r*_3_} is still the optimal solution in the Boolean model. Thus, the Boolean model is more robust for the lack of information in the downstream of a flux, and there is a tradeoff between the accuracy of the model and the risk of the overfitting.

In the flow models, if free variables are introduced or inequalities for the stoichiometric constraint are allowed for internal nodes, the flow model may become more robust for the incorrectness of the distant nodes. However, in such a case, there may be a risk that constraints are not appropriately propagated.

The main difference between the FBA model and EM model is as follows. The basic version of the FBA model requires an objective function, and is formalized as a linear program. On the other hand, in the EM model, all EMs are often enumerated and then are utilized in some optimization problem. These suggest that the FBA model needs an objective function on some target flow(s), whereas the EM model (and also the Boolean model) does not. Therefore, it is suggested that the FBA model is suitable if the objective function is well-defined, otherwise the EM model and/or the Boolean model might be more suitable.

The basic version of the FBA model is efficiently solvable since the linear programming problem is solvable in polynomial time. However, most optimization problems including MCS are NP-hard. For the EM-based model, since the number of EMs may be exponential to the size of the network, the EM-based methods are not feasible for many optimization problems with large-scale networks. The EM-based methods are useful for analyzing details of the flow-based models. Although the MRC of the Boolean model is also NP-hard, improvements of ILP solvers such as CPLEX lead to the speedup for solving the Boolean MRC.

Here, we briefly discuss the scalability of each method based on several reports on computational experiments using real metabolic network data. Related to the FBA-based MRI, the gap-filler method was applied to completion of the metabolic network in the EcoCyc database (version 15.5), which contained 1888 reactions [12]. For the FBA-based MRC, a more complex version was implemented by SimOptStrain, which simultaneously adds and deletes reactions. Although the number of deleted reactions is limited to 10, SimOptStrain was successfully applied to iAF1260, which includes 2077 reactions and 1039 unique metabolites [6]. For the Boolean-based MRC, a more complex version, MKMN (Multiple Knockout for Multiple Networks), was solved by a fast approximation algorithm for the network with 609 reactions and 622 compounds [17]. The Boolean-based MRI was solved for the network with 150 reactions and 93 compounds [16], where an approximation algorithm may be applicable for larger networks. For the EM-based methods, enumerating all EMs was succeeded for the network with 328 nodes, but failed with 1881 nodes [17]. These results suggest that the FBA-based methods are the most scalable, the Boolean-based methods are modestly scalable, and the EM-based methods are less scalable.

### 6.2 Implementation and Application

Many of the constraint-based methods are realized in a software package called COBRA Toolbox, which works on MATLAB [22]. CPLEX is a software tool for solving many types of constraint programming problems including linear programming, mixed integer programming, quadratic programming, and quadratically constrained programming [4].

Although we separately discussed MRC and MRI in this article, adding and deleting reactions at the same time is a reasonable approach in the metabolic network modification. OptReg identifies reaction activations or inhibitions to suggest up-regulation or down-regulation of genes to overproduce biochemicals [21]. OptStrain firstly adds the minimum set of reactions to maximize the theoretical maximum production, and then maximizes the biochemical production by the bilevel programming where the number of deleted reactions is limited [20]. Different from OptStrain, SimOptStrain simultaneously adds and deletes reactions to maximize the biochemical production and the cellular growth in the outer and inner problems, respectively [6]. Applying existing ideas for MRC and MRI to this problem may be a promising future direction.

The FBA-based methods are often used in the process of reconstructing genome-scale metabolic networks, and industrial metabolic engineering. For example, iJO1366 is an FBA-based genome-scale metabolic network of *Escherichia coli*, and can be used for the simulation of cell growth rate for gene knockouts [18]. iNL403 is a core metabolic network of human brains, and used for the simulation of Alzheimer's disease [15]. In these models, the initial models are constructed from genome-sequences and literatures, and then the modification is often conducted to satisfy the experimental data and/or the connectivity of the network. Efficient production of biofuel using microorganisms with metabolic engineering is another important application [13].

For the Boolean model, measures of the impact of knockouts were studied for finding novel drug targets and/or crucial genes for diseases. For example, [14] studied the effect of deletion of each enzyme in the metabolic network of a Boolean model, and [27] considered almost the same problem from the viewpoint of the Boolean aspect of the flux balance mode. Formalizing an optimization problem as MRC with such measures may be useful for finding a gene set as a drug target and/or factors of diseases.

## Figures and Tables

**Table 1:** 5 elementary modes (EMs) of a metabolic network of Fig. 3 when every coefficient of chemical reaction formula is assumed to be 1.

	*r*_1_	*r*_2_	*r*_3_	*r*_4_	*b*_1_	*b*_2_	*b*_3_	*b*_4_
EM1	1	0	1	0	1	1	0	1
EM2	0	0	0	1	0	0	1	1
EM3	1	− 1	1	0	0	1	1	1
EM4	0	1	0	0	1	0	− 1	0
EM5	0	1	0	1	1	0	0	1
